# Vehicle rescue path planning for urban traffic waterlogging disaster based on GAST model

**DOI:** 10.1371/journal.pone.0332989

**Published:** 2025-10-03

**Authors:** You Xiao, Wenjun Cai, Jianan Chen, Chunhui Zhang

**Affiliations:** 1 School of Law and Economics, Wuhan University of Science and Technology, Wuhan, China; 2 Research Center of Public Safety Engineering and Management, Institute of Advanced Studies in Humanities and Social Sciences, Wuhan University of Science and Technology, Wuhan, China; National University of Sciences and Technology NUST, PAKISTAN

## Abstract

Rescue transportation will be severely hindered under urban waterlogging disasters. To solve the problem of delayed rescue of disaster vehicles, this study is based on a hydrodynamic model and uses a 2D hydrodynamic model to numerically analyze urban waterlogging. At the same time, a 1D pipeline network is used to calculate pipeline hydrodynamics and urban hydrodynamics modeling is completed by coupling 2D hydrodynamics with a 1D pipeline network. Considering the characteristics of urban transportation, static and dynamic path planning techniques are introduced to complete vehicle rescue path planning. In the analysis of hydrodynamic models, the monitoring value of the research model at the B water accumulation location was 41.2 cm, which was closer to the actual value and had a lower relative error, outperforming similar models. In addition, in the analysis of multiple rainfall scenarios, the proportion of short-term waterlogging caused by high-intensity rainfall was relatively high. For example, in scenario 4, the proportion of waterlogging formation within half an hour was 8.8%, which was higher than that of low rainfall. In addition, in static path planning, the research technique took 821s in scenario 4 and had a shorter planning distance, which is superior to similar techniques. In dynamic programming, the research technique performed better overall with a driving time of 1,054s in scenario 5 and a shorter planning distance of 7,723m. The research technology has good application effects in urban waterlogging disaster rescue. This research will provide technical support for urban disaster analysis and rescue.

## 1. Introduction

In recent years, frequent weather disasters, especially floods, have posed severe challenges to urban transportation [1]. In this context, it is crucial to effectively support disaster areas and carry out rescue activities [2]. At present, many experts have researched urban disaster models, aiming to provide timely disaster relief in the event of a disaster. Lian J et al. conducted research on rainwater atomization caused by high dam flood discharge. It validated the improved random splashing mathematical model under different bucket shapes and drainage conditions through experiments. They also analyzed the sensitivity of downstream rainfall intensity distribution to multiple factors such as water flow trajectory shape and drainage flow rate. This model had good application effects [3]. Huang et al. attempted to reveal the impact of sediment transport on watershed-scale floods. This study proposed a 2D coupled shallow water hydrological sediment morphology dynamics model. This model was based on the finite volume method and parallel computing on unstructured grids, simulating floods caused by rainfall with different recurrence periods in the Shaanxi Basin of China. The results clearly indicated that sediment transport had an impact on high-intensity current floods, providing important references for watershed flood research [4]. Wang et al. used the InfoWorksI CM hydrological and hydrodynamic model to simulate the depth of urban waterlogging to analyze the impact of landscape patterns and terrain on urban waterlogging. Using it as the dependent variable, under consistent surface and meteorological conditions, they employed Pearson correlation analysis and stepwise regression models for analysis. It has been found that the percentage of built-up areas and urban green spaces has the greatest impact on waterlogging. Therefore, it was necessary to reasonably arrange the patch size of built-up areas, integrate green spaces, and configure terrain gradients to improve waterlogging. This study provided guidance for alleviating urban waterlogging [5]. Zhang Z et al. believed that flood hazards require advance prediction, and therefore proposed a multi-strategy model flood prediction framework. This framework was based on time series prediction and machine learning regression methods, integrating historical rainfall and flood depth, and then proposing an extended rainfall model to select appropriate prediction strategies and optimal model parameters. The selected model had high computational efficiency and was superior to similar models [6].

Under urban flood conditions, vehicle traffic is severely restricted, and ensuring the safe passage of disaster vehicles is a key concern for people. It is necessary to ensure the smoothness of the road and timely delivery of materials within the specified time to meet the requirements of disaster relief. Related scholars have conducted relevant research on path planning [7]. Morin M et al. conducted research on the efficient search path problem in search and rescue operations. The research technology was based on ant colony optimization algorithm variants and search theory, maximizing the probability of finding mobile search objects within limited time and resources. The optimal configuration insights were obtained through experimental analysis, indicating that the technology has good path-planning effects and provides a basis for the implementation of search and rescue path optimization [8]. Zhao J et al. studied the path planning problem for unmanned aerial vehicle base station search tasks. They proposed a dual deep Q-network algorithm that combines state segmentation and optimal state, which classifies and stores multidimensional state information and references received signal strength indicators. They also used a simulation platform to construct a task system model. This scheme could plan the optimal path faster and has advantages in stability and convergence speed [9]. Wang W et al. explored the path planning problem for unmanned aerial vehicle monitoring of geological hazards. To address this issue, the study proposed a heuristic algorithm based on adaptive large neighborhood search. The algorithm results could select necessary monitoring points and access more selectable points within a limited flight time, which has been demonstrated and verified through real cases [10]. Yu Z et al. proposed a hybrid particle swarm optimization algorithm for automatic rescue path planning and to solve the problem of untimely vehicle rescue, and combined it with a simulated annealing algorithm to improve the global optimal solution update strategy. Particles integrated optimal solution information based on dimensional learning strategies. This algorithm could quickly plan high-quality paths and have better robustness in complex 3D environments [11].

The above research indicates that flood disasters have a serious impact on buildings and road traffic, and the hydrodynamic model can provide important data support for regional rescue and disaster prevention through the analysis of rainwater flow. However, the current common hydrological dynamic models have many shortcomings in urban flood analysis, such as hydrological models, rainstorm, and flood management models, which are only applicable to a single traffic scenario and have low calculation accuracy [12]. To meet the requirements of road planning for urban waterlogging vehicle rescue in China and solve the problem of vehicle rescue, this study constructs an urban traffic hydrodynamic model based on the Graphics Processing Unit Accelerated Surface Water Flow and Transport Model (GAST) with Chinese characteristics to complete vehicle path planning. There are two innovations in the research. One is to use the GAST model to model urban rainfall and drainage flow, providing accurate road data for regional rescue. Secondly, based on the characteristics of rescue, static and dynamic path planning methods are introduced to ensure the effectiveness of disaster vehicle rescue. The research content will provide technical reference for road condition analysis and rescue of regional flood disasters.

This study consists of four main parts. The introduction section explores the latest relevant technologies and fields, studies the impact of floods on urban transportation, summarizes the research results of hydrodynamic models and path planning, points out the shortcomings of current models in urban flood analysis, and introduces research techniques based on the GAST model. The method and materials section constructs an urban transportation hydrodynamic model based on the GAST model, including two-dimensional hydrodynamic modeling, one-dimensional pipe network modeling, and model coupling. It also establishes a disaster vehicle path planning model based on waterlogging, introducing static and dynamic path planning methods. The results section analyzes the effectiveness of the hydrodynamic model and the path planning effect under different rainfall scenarios. The discussion and conclusion section summarizes the research results, points out shortcomings, and looks forward to the future.

## 2. Methods and materials

### 2.1. Urban traffic hydrodynamic modeling based on gast model

**Fig 1:**
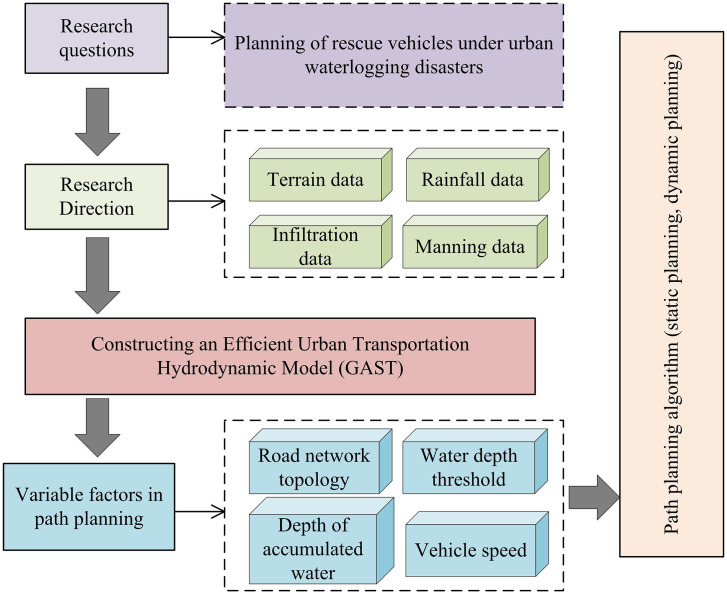
Urban transportation hydrodynamic model framework.

Due to insufficient urban drainage engineering and extreme weather conditions, urban flooding has become increasingly prominent in recent years, posing great challenges to urban transportation [13]. For example, urban waterlogging can exacerbate the damage to road systems and affect the development of urban disaster vehicle rescue work. To solve the above problems, this study is based on GAST and establishes a hydrodynamic model for urban waterlogging, providing data reference for disaster vehicle path planning. The urban traffic hydrodynamic model framework based on GAST is shown in Fig 1.

The model construction process in Fig 1 mainly includes three steps: 2D hydrodynamic modeling, 1D pipeline network modeling, and model coupling. The 2D hydrodynamic model mainly models and analyzes urban rainfall and water flow process control, mainly considering the rainfall loss caused by infiltration, filling, evaporation, and plant interception in the rainfall runoff process [14]. In the analysis of rainfall loss caused by filling in depressions, this study uses the Green Ampt model for infiltration analysis. It can accurately capture the flow characteristics of rainwater filling and the underwater seepage velocity f , measured in mm/min, as calculated in equation (1).


$$f=K_{s}\left(\frac{d+L_{s}+\varphi_{s}}{L_{s}}\right)$$ (1)

In equation (1), d is the depth of surface water accumulation in the soil, measured in millimeters. $K_{s}$ is the hydraulic conductivity, measured in millimeters per minute. $\varphi_{s}$ is capillary suction, measured in millimeters. $L_{s}$ is the depth of the wetting front, measured in millimeters. For the loss of rainfall intercepted by plants, the geographic information system model is used as the basis, and the accumulated amount of plant interception is $S_{\nu }$, measured in millimeters, as shown in equation (2).


$$S_{v}=S_{max}\left[1-e^{-\eta \frac{P_{cum}}{S_{max}}}\right]$$ (2)

In equation (2), $P_{cum}$ is the cumulative precipitation and $S_{max}$ is the maximum interception, both in millimeters. η is the correction factor. The next step is to calculate the net rainfall intensity i: S is defined as the interception rate, I is the rainfall intensity, both in mm/h, used to reflect the remaining rainfall, as shown in equation (3) [15].


$$i=max\left(I+Q_{in}-F-S-E,0\right)$$ (3)

In equation (3), F is the infiltration intensity, $Q_{in}$ is the inflow intensity, and E is the evaporation rate, all in mm/h. After completing the calculation of rainwater flow, this study introduces a 2D shallow water equation to calculate the surface runoff process of rainwater, and the conservation expression is shown in equation (4) [16].


$$S=\frac{\partial q}{\partial t}+\frac{\partial f}{\partial x}+\frac{\partial g}{\partial y}$$ (4)

In equation (4), q is a variable vector. f and g are flux vectors in the y and x directions. The source term vector is S. t is time, measured in seconds. x and y represent the x and y directions. The calculation process of surface runoff of rainwater needs to consider the problems of discontinuous and sudden changes in water flow. In order to effectively solve the conservation expression, an approximate Riemann approach was used for complex fluid solutions of unit momentum and mass flux [17]. The Godunov method solves the local Riemann problem. When combined with the HLLC approximate solver, it effectively captures shock waves and turbulence (such as road water surges) in the two-dimensional shallow water equation, ensuring the conservation of mass and momentum. Compared with the traditional SWMM model, the Godunov method has faster solution efficiency and higher accuracy. Introducing Godunov to discrealize the volume values can improve the computational stability. The integral calculation is shown in equation (5).


$$\int {\mathrm{\backslash nolimits}}_{\Omega }\frac{\delta q}{\delta t}d\Omega +\int {\mathrm{\backslash nolimits}}_{\Omega }\left(\frac{\delta f}{\delta x}+\frac{\delta g}{\delta y}\right)d\Omega =\int {\mathrm{\backslash nolimits}}_{\Omega }Sd\Omega$$ (5)

In equation (5), Ω is the grid control body. Subsequently, equation (5) is adjusted using Gaussian Green’s calculation, as shown in equation (6).


$$\int {\mathrm{\backslash nolimits}}_{\Omega }\frac{\delta q}{\delta t}d\Omega +\oint_{\Gamma }F\left(q\right)\cdot nd\Gamma \mathrm{\backslash nolimits}=\int {\mathrm{\backslash nolimits}}_{\Omega }Sd\Omega$$ (6)

In equation (6), n is the external normal unit vector of Γ. F(q)·n is the flux vector. Γ is the boundary of the grid cell. The calculation of flux vector is shown in equation (7).


$$F\left(q\right)\cdot n=\left(\begin{array}{c} {}*20cfn_{x}+gn_{y} \end{array}\right)$$ (7)

Finally, this study uses approximate Riemann to solve F(q)·n, as shown in equation (8).


$$F\left(q\right)\cdot n=\{\begin{array}{cc} {}*20lF^{L}, & 0\leq S^{L},\\ {F_{*}}^{L}, & S^{L}<0\leq S^{M},\\ {F_{*}}^{R}, & S^{M}<0\leq S^{R},\\ F^{R}, & S^{R}<0 \end{array}$$ (8)

In equation (8), $S_{R}$ is the right side wave velocity. $S_{M}$ is the intermediate wave velocity. $S_{L}$ is the left wave velocity. $F^{L}$, $F_{*}^{L}$, $F_{*}^{R}$, and $F^{R}$ all represent the $F_{k}\left(q\right)\cdot n$ values obtained from different wave velocities. In addition, in the calculation of water flow models, it is necessary to set a time step. This study uses the Coulomb number CFL to represent the time step, as shown in equation (9) [18].


$$\Delta t=CFL\cdot min\left(\frac{R_{i}}{\sqrt{u_{i}^{2}+\nu_{i}^{2}}+\sqrt{gh_{i}}}\right)$$ (9)

**Fig 2:**
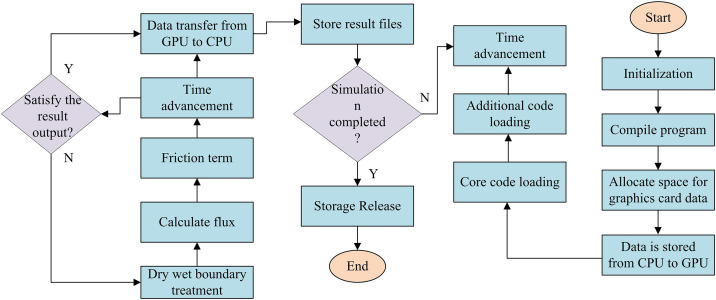
Acceleration calculation process of 2D hydrodynamic model graphics card.

In equation (9), $R_{i}$ is the minimum distance from the boundary to the center of the grid. g is the acceleration due to gravity. $h_{i}$ is the water depth of the unit grid, measured in meters. $u_{i}$ is the unit grid velocity, measured in meters per second. $v_{i}$ is the water flow velocity, measured in meters per second. The range of CFL values is 0<CFL<1. The 2D hydrodynamic model is trained and solved using the NVIDIA CUDA architecture, and the graphics card acceleration calculation is shown in Fig 2.

In Fig 2, the graphics card acceleration process includes compilation, data allocation, storage, simulation, and other processes. Through the graphics card’s internal processing of data, rainfall flow analysis can be achieved.

### 2.2. Urban hydrodynamic coupling modeling based on gast model

In the previous section, numerical calculations of urban stormwater flow were conducted using a 2D hydrodynamic model. To avoid high-intensity rainfall blocking urban traffic, cities mainly discharge rainwater through underground water pipes. Therefore, hydraulic calculations will be performed on the 1D pipeline network, including pipes and nodes, to analyze the water flow of urban drainage pipes through the nodes. However, in heavy rainfall, excessive rainfall can cause blockages in drainage outlets, leading to surface runoff of rainwater [19]. At present, the Storm Water Management Model (SWMM) in the United States is a common pipeline hydrodynamic analysis model, which tends to ignore the characteristics of water surface flow in the analysis of urban rainwater in China [20]. In this study, the dynamic wave method is introduced to analyze the hydrodynamics of pipelines, which can better analyze the water flow state over time, and the dynamic wave method is solved through a coupled 1D pipeline network. The dynamic wave control equation is shown in equation (10).


$$\frac{\partial Q}{\partial x}+\frac{\partial A}{\partial t}=0$$ (10)

In equation (10), A is the cross-sectional area of the water passage, measured in units of $m^{2}$. Q is traffic, measured in units of $m^{3}/s$. The expression for continuing the equation transformation of equation (10) is shown in equation (11).


$$gA\frac{\partial H}{\partial x}+\frac{\partial \left(Q^{2}/A\right)}{\partial x}+\frac{\partial Q}{\partial x}+gAS_{f}=0$$ (11)

In equation (11), H is the fixed cross-sectional water depth, measured in m. $S_{f}$ is the frictional resistance slope. The formula for calculating $S_{f}$ using Manning is shown in equation (12).


$$S_{f}=\frac{K}{gAR^{4/3}}Q\left|V\right|$$ (12)

In equation (12), K is the slope parameter, $K=g{n_{l}}^{2}$, where $n_{l}$ is the Manning coefficient of the pipeline. V is the flow velocity, measured in m/s. R is the hydraulic radius of the water crossing section, measured in m. Based on the above calculation, the node control equation can be obtained as shown in equation (13).


$$\frac{\partial H_{r}}{\partial t}=\frac{\sum_{i=1}^{m}Q_{ti}}{A_{sk}}$$ (13)

**Fig 3:**
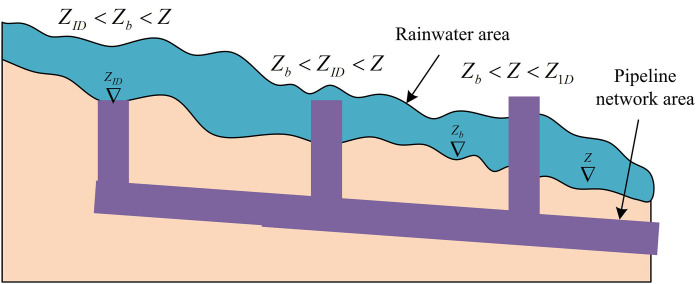
Interaction diagram of three types of water volume in the coupled model.

In equation (13), $Q_{ti}$ is the traffic in and out of the node, measured in units of m/s. $H_{r}$ is the node head, measured in meters. $A_{sk}$ is the base area of the node, measured in $m^{2}$. To effectively analyze the rainwater inlet and outlet pipelines, the pipe network model and hydrodynamic model are coupled for analysis. In coupling analysis, it is necessary to consider the interaction of water volume, which mainly falls into three categories, as shown in Fig 3.

In Fig 3, the water flow interaction mainly includes: when the node water is below the surface layer elevation, referring to node $\overset{Z_{ID}}{\nabla }$, rainwater will flow into the pipeline drainage outlet at the node position. In addition, when the node water is within the range of the surface layer grid water level and the upper layer of the grid, referring to node $\overset{Z_{b}}{\nabla }$ at this moment, the surface water mainly flows into the drainage network from the water head. When the node water is above the surface grid water level, referring to node $\overset{Z}{\nabla }$, the water flow will overflow from the pipeline network to the surface [21]. When rainwater flows into the pipeline network, the calculation of water exchange is shown in equation (14).


$$Q_{in}=\{\begin{array}{c} {}*20l\;m_{1}b\sqrt{2g}{\left(Z\;-Z_{b}\right)}^{\frac{\text{3}}{\text{2}}}\;\left(Z_{1D}<Z_{b}<Z\right)\\ m_{2}A_{node}\sqrt{2g\left(Z-Z_{1D}\right)}\left(Z_{b}<Z_{1D}<Z\right) \end{array}$$ (14)

In equation (14), $Z_{1D}$ and $Z_{b}$ are both node water levels, measured in meters. $m_{1}$ is the low water level orifice flow coefficient. Z is the surface grid water level, measured in meters. $Z_{b}$ is the surface elevation, measured in meters. $Q_{in}$ is the flow rate entering the pipeline network, measured in units of $m^{3}/s$. When rainwater overflows the pipeline network, the calculation of water exchange is shown in equation (15).


$$\begin{array}{c} Q_{out}=m_{3}A_{mode}\sqrt{2g\left(Z_{1D}-Z\right)}\left(\;\;\;\begin{array}{c} {}*20cZ_{b}<Z<Z_{1D} \end{array}\;\;\;\right) \end{array}$$ (15)

**Fig 4:**
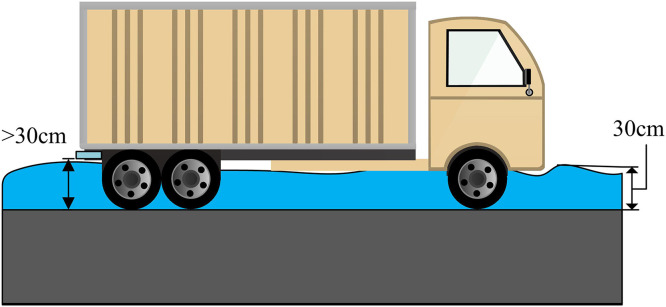
Schematic diagram of vehicle wading depth.

In equation (15), $Q_{out}$ is the flow rate flowing out of the pipeline network, measured in units of $m^{3}/s$. $A_{node}$ is the cross-sectional area of the rainwater outlet, measured in $m^{2}$. $m_{2}$ is the medium flow orifice flow coefficient. In the analysis of urban heavy rainfall and waterlogging disasters, when the amount of rainwater on urban roads exceeds the drainage load standard of the road network, it will cause rainwater to spread on urban roads, forming waterlogging and blocking traffic [22]. When the vehicle is submerged in water beyond its own exhaust port, it will cause rainwater to enter the vehicle and cause it to stall [23]. Specifically, the vehicle’s wading depth value is shown in Fig 4.

The vehicles affected by waterlogging in this study are required to exhaust within the range of 30 cm to 40 cm. If the minimum exhaust height for disaster vehicles is set as the critical wading depth, then the critical wading depth is 30 cm. If the waterlogging on urban roads exceeds 30 cm, vehicles will be prohibited from passing through that section, and the depth of waterlogging on urban roads will be determined through the GAST model.

### 2.3. Modeling of disaster vehicle path planning based on urban waterlogging

**Fig 5:**
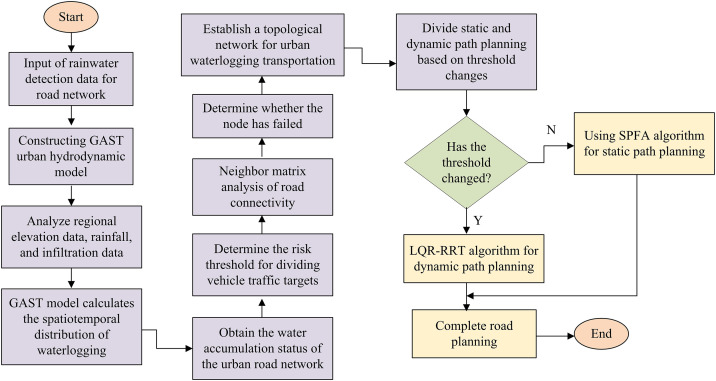
Path planning process for rescue vehicles based on GAST model.

Next, rescue path planning for disaster vehicles will be carried out based on the GAST model. The idea of rescue road planning is to use the GAST model to analyze and judge the distribution of urban road waterlogging and the depth of water involved. Additionally, path planning algorithms are employed to formulate the rescue path for disaster vehicles [24]. The entire disaster vehicle path planning based on the GAST model is shown in Fig 5.

According to the disaster vehicle planning in Fig 5, the road risk is determined based on the analysis of urban road rainfall using the GAST model, and the road planning algorithm is used to analyze water depth and vehicle speed. In the specific planning process, it is necessary to consider the selection of static and dynamic scene technologies, experimentally design two algorithms, and use them separately in two scenarios. Among them, static planning is suitable for building long-term stable frameworks in cities, ensuring the global optimal solution and ensuring the stability of infrastructure. Dynamic programming mainly considers temporal variables such as traffic and rainfall, and implements scheme planning through multi-stage decision models. Complete emergency rescue of urban waterlogging disaster vehicles through dynamic and static planning. In specific analysis, urban roads can be understood as a topological network composed of numerous roads, and road intersections can serve as nodes. In this study, the proximity matrix is used to describe the connectivity of the road network, such as whether the road sections were congested or the pedestrian flow was too high, to provide support for subsequent vehicle rescue planning [25]. The adjacency matrix representation of the road network is shown in equation (16).


$$G=\left[\begin{array}{c} {}*20cN,L \end{array}\right]$$ (16)

In equation (16), L is the set of road network segments. N is a set of road network nodes. The adjacent matrix weight value ω is used in the road section to reflect the traffic capacity of disaster vehicles, as shown in equation (17).


$$\omega =\left[\begin{array}{ccc} {}*20c\omega_{11} & \cdots & \omega_{1n}\\ \vdots & \ddots & \vdots \\ \omega_{n1} & \cdots & \omega_{nn} \end{array}\right]$$ (17)

**Fig 6:**
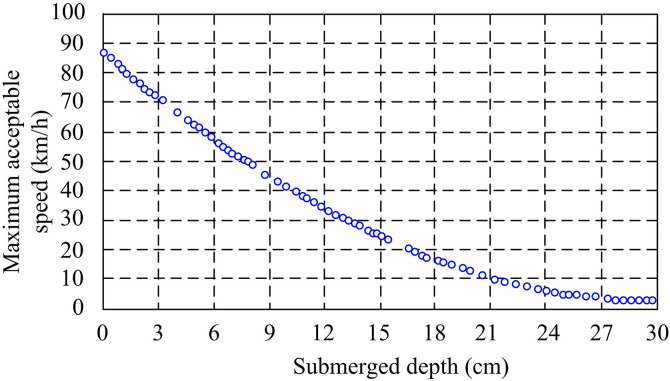
Relationship between water depth and velocity curve.

In specific vehicle road planning, each topology node will change according to the situation of waterlogging, including node or section failure, which requires a threshold range for each section to be determined. If the threshold information remains constant for a certain period, it indicates that the rainwater information has not been updated in real time and is currently in static planning. The change in threshold indicates a change in rainwater information, which is currently referred to as dynamic programming [26]. Static planning studies the relationship curve between water depth and speed on urban roads, taking into account both urban safety and rescue needs. The relationship between water depth and velocity curve is shown in Fig 6.

In vehicle rescue, only the depth of accumulated water and vehicle speed are considered, ignoring factors unrelated to waterlogging. The maximum speed of disaster vehicles is set to 60 km/h. Due to the different water depths in the flooded areas of urban roads, the vehicle speed may change. Therefore, the weight matrix is calculated based on the time the vehicle passes through the road section, as shown in equation (18).


$$t_{m}=\sum_{n=1}^{k}\frac{L_{o}}{v_{n}}\times 3600$$ (18)

In equation (18), o is the number of section m. $t_{m}$ is the travel time under section m, measured in s. k is the total sub section number. $L_{n}$ is the length of sub section n, measured in kilometers. $\nu_{o}$ is the speed of sub section o, measured in units of km/h. Static planning adopts the Shortest Path Faster Algorithm (SPFA) as the static planning strategy. Compared to Floyd Marshall algorithm and Dijkstra algorithm, it has higher efficiency and can handle negative weight edges. The state transition expression of SPFA is shown in equation (19) [27].


arc[i\rightleft[j]=min(arc[i][j],arc[i][x]+arc[x][j]) (19)

In equation (19), x is the waypoint between i and j. arc[i][j] is the shortest distance between i and j, measured in km. In dynamic programming, the weight of road segments will change according to the water accumulation situation on the road, ensuring optimal vehicle planning. At present, there are dynamic path planning algorithms such as Dynamic Programming (DP) and Dynamic A Algorithm (A*), but the overall planning efficiency is average and limited for complex road condition planning [28]. In this study, a Linear Quadratic Regulator – Rapidly-exploring Random Tree (LQR-RRT) is used for dynamic path planning. It is an improved algorithm of the random tree algorithm, with good dynamic adaptability and suitable for complex road conditions [29]. The state network set within 1 hour is denoted as R, expressed as equation (20).


R=(G,T) (20)

In equation (20), T represents the period of change, and G identifies the periodic traffic network as a static network G=[N,L]. At different times, road network G will be adjusted to $G_{l}$, $G_{2}$,..., $G_{T}$. $n_{0}$ is set as the starting point, the endpoint is $n_{d}$, and the connected path is defined as OD [30]. OD is composed of multiple adjacent nodes $\left(n_{0},n_{1},\cdots ,n_{d}\right)$, and the weight expression of OD road segment is shown in equation (21).


$$\omega_{L}=\sum_{n_{i}\in d}\delta \left(n_{i},n_{i+1}\right)=\delta_{\left(t_{0}\right)}\left(n_{0},n_{i}\right)+\delta_{\left(t_{1}\right)}\left(n_{i},n_{i+1}\right)+\cdots +\delta_{\left(t_{m}\right)}\left(n_{i+k},n_{d}\right)$$ (21)

In equation (21), δ is a mapping function that conforms to time variation. m is the time step. $t_{0}$, $t_{l}$,..., $t_{m}$ all represent the time period of change. k is the number of road sections. This study aims to find the dynamic path optimal solution with the minimum cost of OD, as shown in equation (22).


$$\begin{array}{c} \omega_{OD}=\sum_{l\prime \in l}min[\sum_{l\prime \in l}\delta \left(n_{i},n_{i+1}\right){]}_{\left(t\right)} \end{array}$$ (22)

In equation (22), $l^{\prime }$ is a non-repeating segment of path l. ${\left[\cdot \right]}_{\left(t\right)}$ is the path that passes through period t.

## 3. Results

### 3.1. Analysis of urban hydrodynamic model

**Fig 7:**
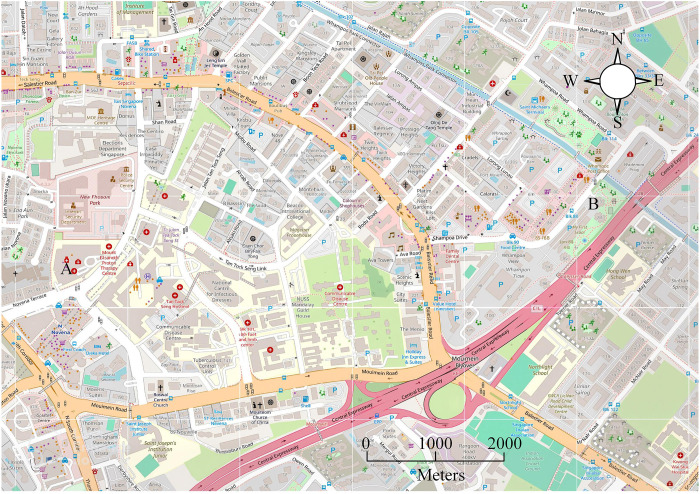
Structure of urban road network in the study area (Source from: https://www.openstreetmap.org/#map=16/1.32562/103.85308).

This study focuses on the main urban area of a city in the central and northern regions, covering an area of 24.25 km2. The building density in this area is high, and there are rivers passing through it, showing a trend of high in the eastern region and low in the western region. The research area has a typical semi humid climate on land, with an average annual rainfall ranging from 531 mm to 660 mm. Urban water environment information: The research area is located in the main urban area of a city in central and northern China (24.25km^2^), belonging to the confluence area of tributaries of the North Canal water system. The river gradient is 0.5%−1.2%, the flow rate during the dry season is 0.8-1.2m^3^/s, and the peak flood during the flood season reaches 80m^3^/s (50 year return period). The average annual rainfall in the region is 531–660 mm, with an evaporation rate of 1120 mm. The groundwater depth is 12-18m, and the permeability coefficient is 1.2 × 10-5m/s. The density of the drainage network is 15.2 km/km2, with a design return period of 3–5 years. The proportion of hardened pavement is 42% (runoff coefficient 0.85), and the comprehensive runoff coefficient is 0.68–0.75. The historical maximum depth of accumulated water is 1.8m (2021), and the dynamic obstacle avoidance radius is set to 3.6m. The water quality COD is 45–60 mg/L, NH 3-N 1.2–2.5 mg/L, exceeding the Class IV standard. The utilization rate of recycled water is ≥ 40%, equipped with a 120000 m^3^ storage tank, which is in line with the hydrological characteristics of high-density built-up areas. The schematic diagram of the urban road network structure in the research area is shown in Fig 7.

**Fig 8:**
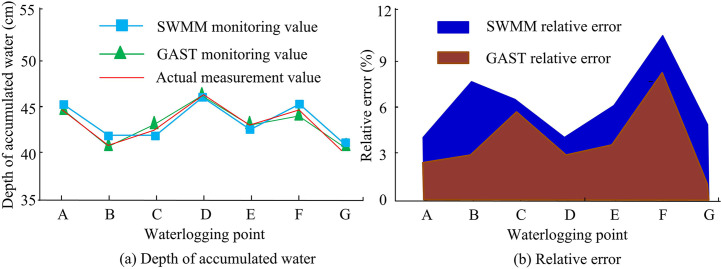
Results of GAST model validity test.

To verify the feasibility of the research technology, the following will take the detection data from the meteorological monitoring center in the region as an example and use Chicago rainfall patterns for simulation calculations. This study uses the Python programming software “NnetworkX” to calculate and analyze the node degree and other information of the roads in the study area. The higher the node degree value, the greater the traffic flow of the road section and the higher the number of roads. The actual rainfall data from July 3, 2020 in the region was selected to test the effectiveness of the GAST model, and the SWMM developed by the US Environmental Protection Agency was introduced as the test object. The specific results are shown in Fig 8.

**Fig 9:**
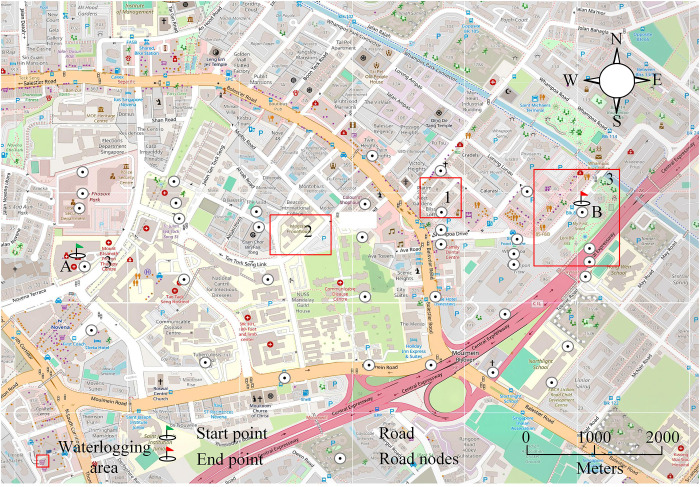
Topological diagram of urban road network structure (Source from: https://www.openstreetmap.org/#map=16/1.32562/103.85308).

Fig 8 shows the comparison results of water accumulation simulations using different hydrodynamic models. In the simulation comparison of waterlogging depth, compared with the monitoring data of SWMM model, the monitoring data of GAST model are significantly closer to the actual value. Seven flooded areas in the research site are selected for testing. At the location of water accumulation in B, the simulated monitoring value of GAST is 41.2 cm, while the simulated monitoring value of SWMM model is 44.2 cm, and the actual value is 41.5 cm. The accuracy of the GAST model is higher. The relative error comparison is based on the flooded area F, where the SWMM model has a relative error of 10.83%, while GAST has a relative error of 8.14%. In addition, the relative error of the simulated monitoring data for the 7 flooded areas is within 8%. Therefore, the GAST model is more accurate in simulating and monitoring waterlogging in the study area, indicating that GAST has high reliability and can meet the requirements of urban waterlogging analysis. Next, this study conducts road planning analysis for rescue scenarios based on the distribution of main roads, where points A and B are the starting and target endpoints for disaster vehicles. According to the severe flood disaster faced by the target point, it is necessary to call materials from point A to rescue the disaster at point B. Fig 9 is a topological diagram of the urban road network structure constructed based on rescue efforts.

**Fig 10:**
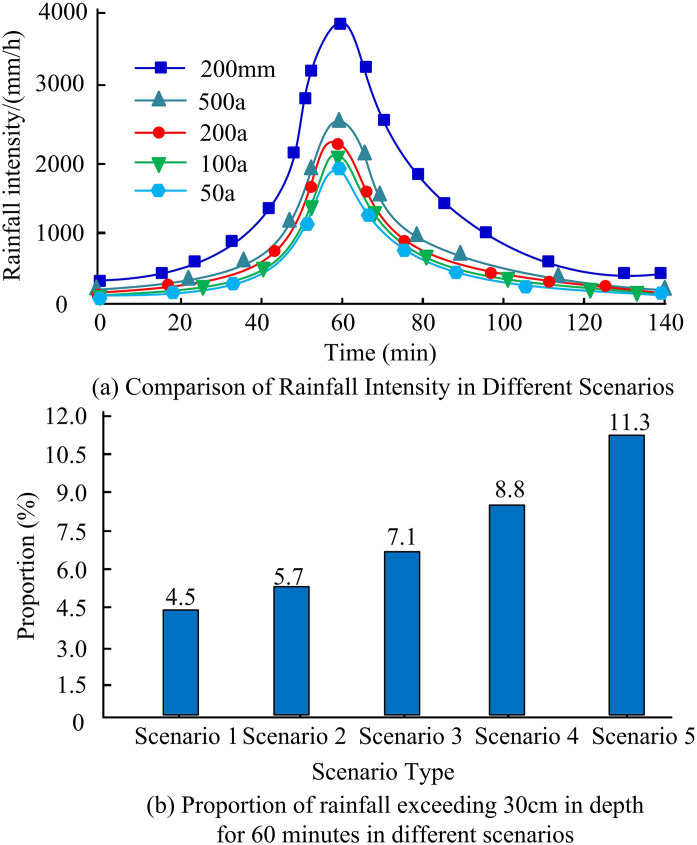
Numerical simulation analysis of 5 different rainfall scenarios.

In Fig 9, this study uses the original method to analyze the roads in the research area and constructs a road network topology diagram. There are a total of 114 nodes and 175 road segments. To simulate and test the waterlogging data in the region more accurately, rainfall scenarios will be set up based on the rainfall data of the region in the past 40 years, including scenario 1 (50 years), scenario 2 (100 years), scenario 3 (200 years), scenario 4 (500 years), and scenario 5 (200 meters). Scenario 1 is an environment without rainfall. Fig 10 shows the simulation results of rainfall intensity within 140 minutes based on 5 types of rainfall scenarios.

**Fig 11:**
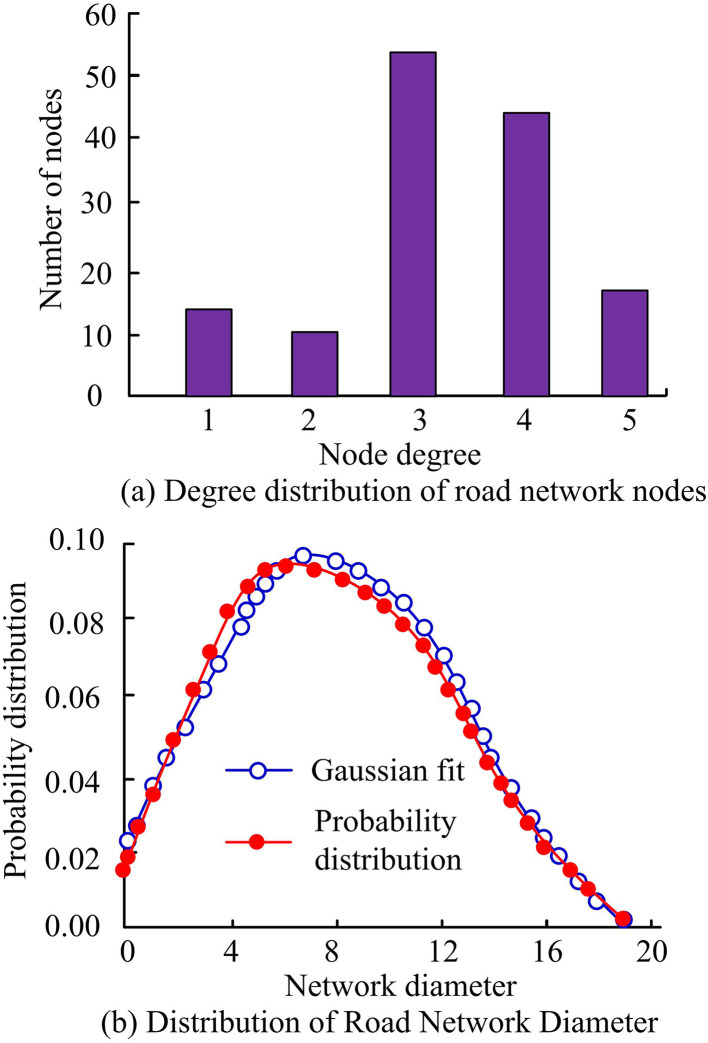
Distribution of overall road node degree and road network diameter in the study area.

In Fig 10a, there are significant differences in rainfall intensity under different scenarios, and with the increase of rainfall time, the rainfall intensity expands significantly and forms urban waterlogging. In scenario 1, around 60 minutes, it reaches its maximum rainfall intensity, with a maximum rainfall intensity of 2,000 mm/h. The proportion of water depths exceeding 30 cm in Fig 10b corresponds to 4.5% in Scene 1. In scenario 4, the proportion of water depths exceeding 30 cm after 60 minutes of rainfall reaches 8.8%, while in scenario 5, the proportion reaches 11.3%. The waterlogging area in Fig 10 shows that waterlogging areas 1 and 2 are located in low-lying areas, with obvious water accumulation in scenarios 4 and 5, and the surrounding roads have become ineffective, making it impossible to continue traffic. The rescue of flood prone area 3 is even more severe, as there are numerous residential buildings in the area and insufficient drainage pipeline capacity, resulting in traffic failure on multiple road sections in the region. Fig 11 shows the results of analyzing the degree and diameter distribution of road network nodes using the complex characteristics of urban road networks and the NetworkX library.

**Fig 12:**
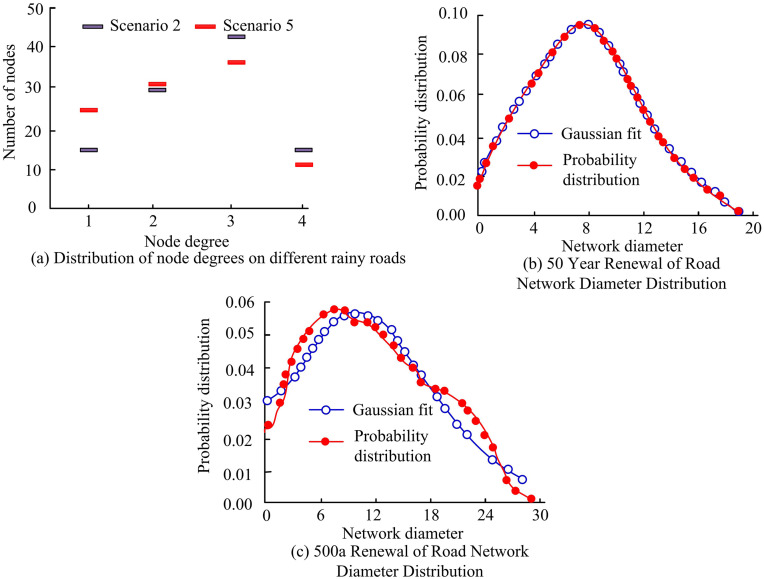
Node degree and diameter distribution of road networks in multiple scenarios.

In Fig 11a, the average node degree in this area is 3.2. When the node degree is 2, it indicates that there is a turning area at that location, and when the node degree is higher than 2, it indicates that the location is a network intersection. The analysis results show that the area is mainly dominated by intersections. Further analysis reveals a phenomenon of increasing node degree and decreasing node count, indicating the presence of super nodes in the region, resulting in a complex road network. Subsequently, the diameter distribution of the road network is studied using Gaussian fitting function. In Fig 11b, the road network fitting degree is above 90%, and the highest probability distribution network diameter is 7.1, indicating that the mean shortest path is 7.1. To further analyze the distribution characteristics of the road network, scenarios 2 and 5 are introduced for analysis, as shown in Fig 12.

Fig 12 shows that as the rainfall scenario intensifies, areas with higher node density will experience a decrease in the number of nodes due to an increase in rainfall intensity (resulting in network failure in some road segments). The failure rates of the road network in scenarios 2 and 4 are 23.2% and 34.9%. By using Gaussian fitting function to analyze the diameter distribution in two scenarios, the fitting degree in scenario 4 is less than 80%, and the increase in road failure rate actually leads to a decrease in the complexity of the road network.

### 3.2. Analysis of vehicle path planning for urban flood disaster

**Fig 13:**
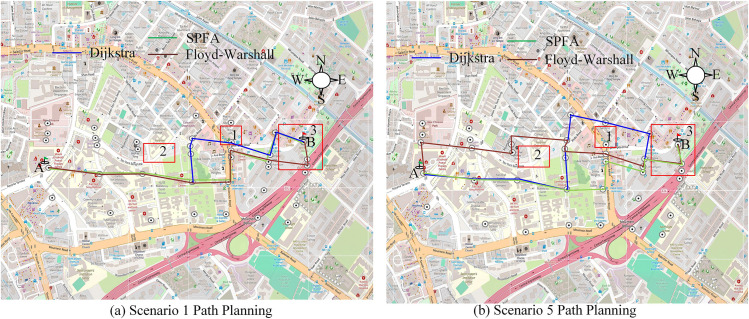
Static path planning (Source from: https://www.openstreetmap.org/#map=16/1.32562/103.85308).

Next, this study conducts path planning analysis for disaster vehicles based on the GAST model and completes path planning simulation analysis through Python. When the short-term rainfall intensity in the city reaches 50–100 mm/h, low-lying areas and road sections with a high proportion of hardened roads are prone to water accumulation with a depth exceeding 20 cm, which can restrict vehicle traffic. In addition, when urban areas face rainfall of over 100 mm/h, the capacity of urban drainage networks will be limited, and traffic in tunnels and low-lying areas will be severely affected. According to the discovery of high-intensity rainfall in urban areas over the years, high-intensity rainfall can easily lead to overloading of urban drainage networks, causing water accumulation at key intersections, overpasses and other transportation hub nodes. Road traffic is prone to risks such as failure of some key transportation nodes, secondary disasters caused by pipeline overflow, inaccessible paths in low-lying areas, and delayed dynamic data updates, which have a serious impact on emergency vehicle rescue. Therefore, the following research will consider multiple scenarios for planning. Among them, path planning is divided into static path planning and dynamic path planning based on the different threshold parameters of the hydrodynamic model. In actual planning, vehicles avoid flooded areas and long red light areas based on real-time road condition information and GAST model feedback, striving to reach the target point with the shortest time and distance. This study selects typical no rainfall scenario 1 and high-intensity rainfall scenario 5 for static path planning, as shown in Fig 13.

**Fig 14:**
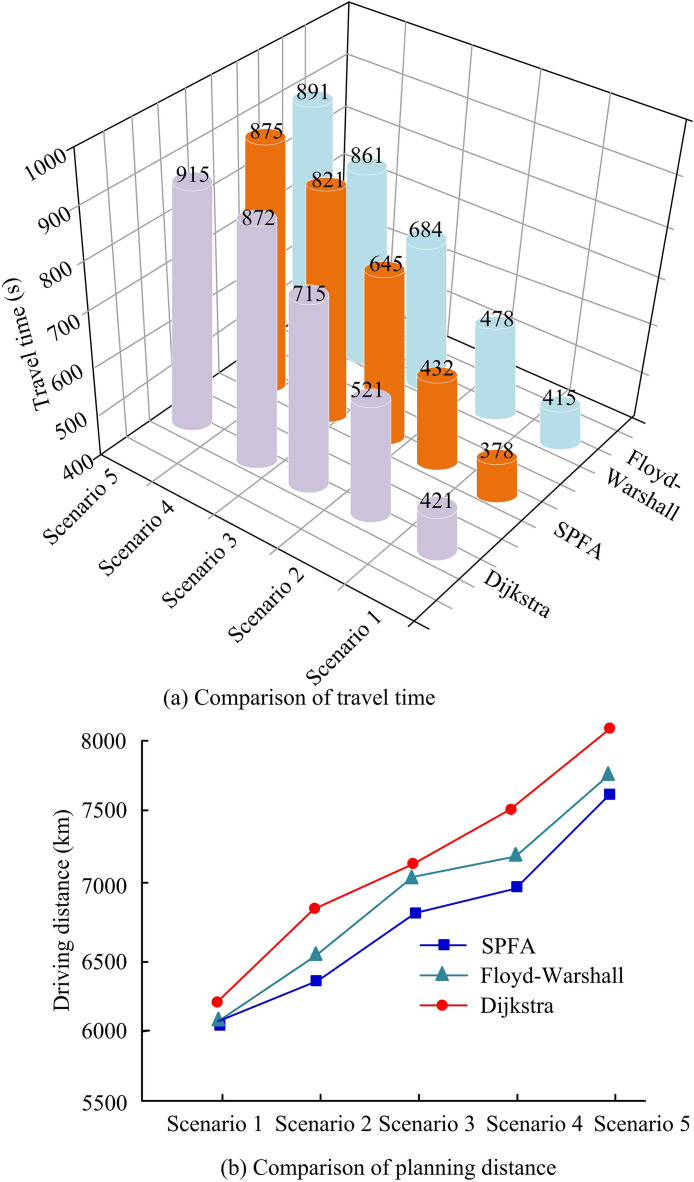
Comparison of travel time and planned distance under static path planning.

In scenario 1 without rainfall in Fig 13, the planning distances of the three planning models are generally close, but the Dijkstra model passes through the flood prone area 1 in the planning. There are multiple intersections and traffic lights in this area, resulting in a longer overall planning time. In scenario 5 planning, based on the feedback of water accumulation from the GAST model, the SPFA model prioritizes the goal of avoiding flooded areas with fewer red light stops and shorter planned paths, which has significant advantages compared to Dijkstra and Floyd-Warshall. Dijkstra’s plan chooses to detour around flooded areas, resulting in an overall longer planned distance. Floyd-Warshall passes through multiple intersection nodes during planning, resulting in longer stopping times at traffic lights compared to SPFA. The static planning of different models in multiple scenarios is shown in Fig 14.

**Fig 15:**
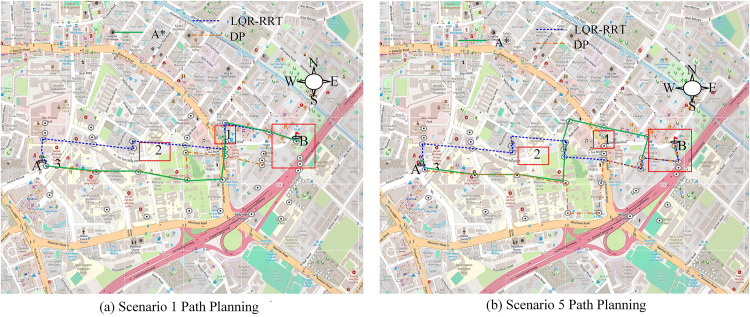
Dynamic path planning (Source from: https://www.openstreetmap.org/#map=16/1.32562/103.85308).

Fig 14a shows a comparison of formal time. The SPFA model is significantly better than similar models, with SPFA taking 821s, Floyd-Marshall taking 861s, and Dijkstra taking 872s in scenario 4. In other scenarios, SPFA still has good planning advantages compared to other models. In the comparison of planning distances in Fig 14b, the overall planning distance of SPFA is shorter. For example, in Scenario 3, the planning distances of SPFA, Floyd-Marshall, and Dijkstra are 6,885m, 7,123m, and 7,235m, respectively. In scenarios 1, 2, 4, and 5, SPFA still maintains a good planning advantage, reducing detours and crossings while avoiding flooded areas, resulting in the best overall planning effect. Fig 15 shows the results of dynamic programming.

**Fig 16:**
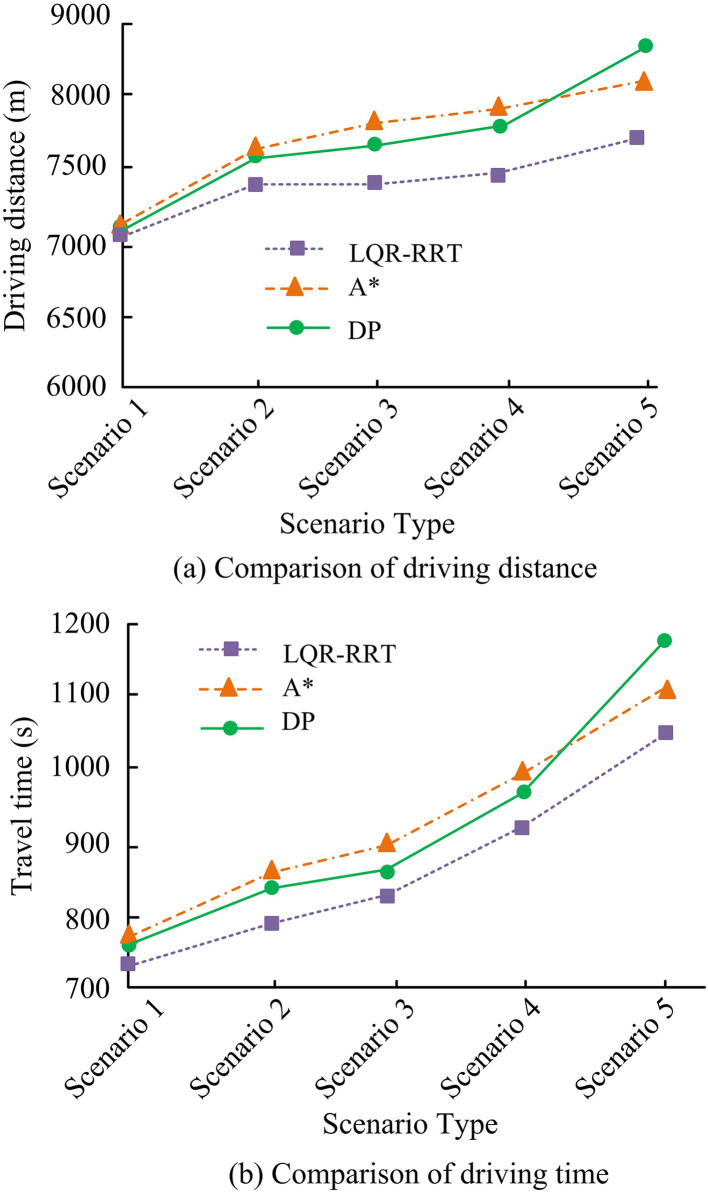
Comparison of travel time and planned distance under dynamic path planning.

In scenario 1 without rainfall in Fig 15, both models prioritize selecting short distances as the objective. Due to the minimal impact of rainfall, the planning distances of the three models are close. However, LQR-RRT prioritizes avoiding intersections and waiting for traffic lights in planning, so the overall planning time is relatively shorter. In scenario 5 with heavy rainfall, multiple areas experience waterlogging, and all three dynamic programming models show repeated planning. After bypassing the flooded area 2, A* chooses to bypass the flooded area 1 again, resulting in multiple increases in the overall planning length. DP chooses to detour from below the flooded area 2 in the planning, but due to excessive rainfall, it has to change its route, resulting in a significant increase in overall time and planning length. The specific plan is shown in Fig 16.

Fig 16a shows a comparison of driving distances. In scenario 1 and scenario 2, the three models have close planned distances. All three models can dynamically select the shortest distance for rainfall hours, but the LQR-RRT model prioritizes fewer intersections and stopping areas in planning, resulting in better overall planning. In scenario 5 with severe waterlogging, the driving distances of LQR-RRT, A*, and DP are 7,723m, 8,125m, and 8,845m, while the driving times are 1,054s, 1,112s, and 1,190s, respectively. Overall, LQR-RRT has better efficiency and planning distance in dynamic programming. Finally, the study re selected new roads (from point A to point C) from the urban road network and tested them in different rainfall scenarios. Among them, the time for the vehicle to be in a clear state from point A to point C is 1325 seconds, and the planned shortest distance is 9856 meters. In this study, four rainfall scenarios with rainfall of 200 mm and rainstorm repetition period of 50 years, 100 years and 200 years were selected for vehicle rescue path planning test. The results are shown in Table 1.

**Table 1:** Comparison of rescue vehicle planning in multiple rainfall scenarios.

Different scenarios	Method	LQR-RRT		A*		DP	
	Number of experiments	Planning time (s)	Planning distance (m)	Planning time (s)	Planning distance (m)	Planning time (s)	Planning distance (m)
50a	1	1758	10250	1895	11587	1984	11682
	2	1748	10350	1872	11456	1905	12584
	3	1754	10215	1924	10975	1905	11025
	4	1761	10312	1864	10998	1978	10985
100a	1	1856	10583	1954	10685	2025	11545
	2	1893	10756	2045	10698	1984	12586
	3	1985	10658	2145	10605	2105	10985
	4	1874	10256	2021	17515	2098	11256
200a	1	2154	12456	2345	25463	2375	26452
	2	2234	12756	2394	26012	2404	27025
	3	2164	11954	2456	24126	2394	26458
	4	2245	12974	2345	25145	2504	27545
200mm	1	2345	13456	2645	16452	2894	16785
	2	2464	14245	2754	16484	2945	17041
	3	2386	13846	2675	15945	2845	17284
	4	2458	14854	2795	16785	2759	15894

According to the test results in Table 1 the LQR-RRT model has a more stable planning effect in various rainfall scenarios, with a planning time and distance close to each other after four repeated tests. The DP model has significant differences in planning after multiple tests. In addition, the planning time and distance of LQR-RRT in the same precipitation scenario are significantly lower than those of the A* model and DP model. In a 200 mm rainfall scenario, the average planned consumption of LQR-RRT is 2,413.25s and the average planned distance is 14,100.25s. The average planning time for A* is 2,717.25 seconds and the average planning distance is 16,416.50 meters. DP performs the worst, with an average planning time of 2,860.75s and an average planning distance of 16,751.00m, respectively. Next, the study introduced Friedman test and Wilcoxon signed rank test to analyze the planning effects of different techniques, and the test results are shown in Table 2.

**Table 2:** Statistical analysis of data verification for different technical planning.

Scenario	50a		100a		200a		200mm	
Metric	Planning time	Planning distance	Planning time	Planning distance	Planning time	Planning distance	Planning time	Planning distance
LQR-RRT (Mean Rank)	1.2	1.1	1.3	1.2	1.1	1	1.4	1.3
A* (Mean Rank)	2.3	2.4	2.2	2.3	2.4	2.5	2.1	2.2
DP (Mean Rank)	2.5	2.5	2.5	2.5	2.5	2.5	2.5	2.5
Friedman χ²	9.84	11.25	8.97	10.73	12.06	13.82	7.95	9.21
p-value	0.007	0.004	0.011	0.005	0.002	<0.001	0.019	0.01
p*	0.013	0.008	0.019	0.012	0.005	0.002	0.028	0.017
p**	0.011	0.006	0.009	0.007	0.003	0.001	0.015	0.012

Note: p* represents the comparison between LQR-RRT and A*; P** represents the comparison between LQR-RRT and DP.

According to the statistical test results in Table 2, in Planning Time, the Friedman test *p* < 0.05 for all scenarios (e.g., *p* = 0.002 for scenario 200A), indicating that there are essential differences in time efficiency among the three algorithms. In the Planning Distance test, *p* < 0.001 was found in the 200A scenario, indicating a significant difference, which suggests that the algorithm has an undeniable differentiation in its ability to optimize path length. Further analysis revealed that the average ranking of LQR-RRT in the time/distance metric remained stable at 1.0–1.4, significantly lower than A* (2.1–2.5) and DP (2.5), indicating better overall performance. For example, in terms of algorithm stability, the planning time standard deviation of LQR-RRT (50 scenario σ = 5.2) is significantly lower than A * (σ = 24.3) and DP (σ = 38.7), verifying its strong adaptability to complex environments. From this, it can be seen that the research model has better stability and planning effectiveness under different rainfall scenarios. Finally, the study selected the central urban area of Yanta District in Xi’an City for actual road condition testing, with an area of 29.46 km2. The area has a high building density (plot ratio of 2.8), a road network density of 15.2 km/km^2^, and a historical maximum waterlogging depth of 1.8 meters, indicating typical urban waterlogging characteristics. The GAST-SWMM coupling model was used to construct the 1D-2D hydrological and hydrodynamic model, with a grid accuracy of 5m × 5m and dynamically adjusted time steps. The success rate of path replanning for multi scenario testing algorithms was set as shown in Table 3.

**Table 3:** Success rate of multi scenario path replanning.

Test scenario	SPFA (Static)	LQR-RRT (Dynamic)	A*
Regular commuting	98.70%	96.40%	93.20%
Local waterlogging (30 cm)	92.10%	89.70%	84.30%
Global waterlogging (50 cm)	76.50%	82.30%	68.90%
Pipeline overflow event	Unable to respond	78.40%	62.10%

According to the results in Table 3, the study set up four scenarios and tested three algorithms. In dynamic scenarios such as pipeline overflow, the path replanning ability of LQR-RRT increases the success rate by 28.6% compared to static SPFA, highlighting the emergency adaptability of dynamic algorithms. On the other hand, static algorithms can maintain their advantages over traditional A * in addition to pipeline overflow events, indicating that the proposed technology has good cross scenario robustness.

## 4. Discussion and conclusion

In recent years, with the intensification of climate change, extreme weather conditions have gradually increased, and many cities have experienced abnormal flood disasters. High-intensity rainfall can increase the load on urban drainage pipelines in a short period, leading to waterlogging and large-scale waterlogging, posing significant challenges to urban safety and transportation. To meet the requirements of urban rescue for disaster vehicles, this study is based on the GAST model to analyze the urban rainfall patterns and distribution and establish a hydrodynamic model. Meanwhile, static and dynamic path planning techniques are introduced based on regional hydrodynamic analysis data, effectively achieving the goal of disaster rescue vehicle path planning and providing support for regional disaster rescue.

Seven typical urban waterlogging areas were selected for water depth simulation monitoring through experimental analysis. In area B, the GAST simulated monitoring value was 41.2 cm, the SWMM model was 44.2 cm, and the actual value was 41.5 cm. The simulation monitoring results of GAST were closer to the actual values, and compared with SWMM, the monitoring relative errors in the 7 areas were significantly lower. This indicated that the GAST model had good application effects and was more suitable for urban rainfall analysis in China. In addition, five different rainfall scenarios were selected for the complexity analysis of urban road networks in the study area. Further increase in high node degree led to a decrease in the number of nodes, such as when the node degree increased from 3 to 4, the number of nodes significantly decreased, indicating the presence of super nodes in the region. Meanwhile, areas with high node density might experience a decrease in the number of nodes due to an increase in rainfall intensity, leading to the failure of transportation networks in some regions. The reason for this phenomenon was that high-intensity rainfall blocked drainage pipes and caused waterlogging in the area, hindering traffic. Meanwhile, according to the Gaussian fitting function, the Gaussian fitting degree of high-intensity rainfall scenario 4 was less than 80%, and the failure of the road network reduced its complexity.

Based on the analysis of the urban road network in the research area, static and dynamic path planning scenarios were divided. In static planning, the SPFA model had a planning distance of 6885m and a time of 821s in scenario 3, which was superior to Floyd-Marshall and Dijkstra in terms of planning time and distance. In static path planning, the SPFA model had better time complexity and higher algorithm efficiency, which was significantly better than similar models. In dynamic path planning, the driving distances of LQR-RRT, A*, and DP in scenario 5 were 7723m, 8125m, and 8845m. The driving time of LQR-RRT was significantly shorter, at 1054s, and the overall efficiency and planned distance of LQR-RRT were better. The main reason was its ability to quickly explore space and generate paths, and it could also handle high-dimensional and complex constraints well.

In summary, this study analyzed road traffic under urban waterlogging scenarios and established a hydrodynamic model. The experimental results show that the GAST model has good application effects. Overall, this technology is suitable for medium-sized cities with built-up areas ranging from 20–100km^2^. The urban functions of such cities are relatively concentrated, the drainage network scale is easy to manage, the hydrological boundaries are clear, and it is convenient to achieve the coupling of one-dimensional pipe networks and two-dimensional surface hydraulic systems. Meanwhile, in terms of terrain, technological adaptation requires relatively concentrated urban functions, easy management of drainage network scale, clear hydrological boundaries, and ease of coupling one-dimensional pipeline networks with two-dimensional surface hydraulic systems. In addition, technology also has certain requirements for meteorology in its application, mainly suitable for cities with temperate monsoon climate or subtropical humid climate, where rainfall is concentrated in specific seasons, such as regional annual rainfall of 500–1200 mm, of which 60% −80% of rainfall is concentrated in 3–5 months. The rainfall patterns in these areas are relatively regular, which is conducive to the calibration of hydrodynamic model parameters. However, research techniques rely on specific urban hydrological information data and have poor adaptability to cities with scarce hydrological and infrastructure data. For smaller cities with built-up areas less than 5km^2^, their drainage systems are highly dependent on the surrounding natural water systems, resulting in a “boundary effect” problem that the hydrodynamic model cannot adapt to. In addition, the layout of urban water systems is irregular, and cities with damaged surface drainage systems find it difficult to adapt. In the future, improvements will be made in three aspects: firstly, establishing a multi-source data fusion framework, integrating unmanned aerial vehicle remote sensing, IoT monitoring, and historical hydrological data, constructing a dynamic parameter adaptive correction model, and using data filling algorithms to compensate for the lack of hydrological and infrastructure data; Secondly, for small-scale cities with a built-up area of less than 5km^2^, the boundary condition setting method of the model is optimized, and the interaction coefficient of surrounding natural water systems is introduced to quantify the impact of “boundary effects” on the drainage system, thereby improving technical adaptability; The third is to incorporate urban spatial form characteristic parameters, which can increase the topology reconstruction algorithm for irregular water system layout, simulate the variation of water flow path after surface drainage system damage, and thus improve technical adaptability.

## Supporting information

S1 File Minimal data set definition. (DOC)
