# Supplementary material for: Vehicle rescue path planning for urban traffic waterlogging disaster based on GAST model
Source: PLoS One. 2025 Oct 3;20(10):e0332989. doi: 10.1371/journal.pone.0332989 (PMC12494290; doi:10.1371/journal.pone.0332989)
Supplement: S1 File — (DOC) [file pone.0332989.s001.doc]

**The data in Figure 8 (a)**

| Method | A(cm) | B(cm) | C(cm) | D(cm) | E(cm) | F(cm) | G(cm) |
| --- | --- | --- | --- | --- | --- | --- | --- |
| SWMM | 45.5 | 42.5 | 42.8 | 46.5 | 43.5 | 45.8 | 42.8 |
| GAST | 45.3 | 41.5 | 43.8 | 46.2 | 43.2 | 44.5 | 42.5 |
| Actual | 45.2 | 41.4 | 43.2 | 46.3 | 43.1 | 44.9 | 42.4 |

**The data in Figure 8 (b)**

| Method | A(%) | B(%) | C(%) | D(%) | E(%) | F(%) | G(%) |
| --- | --- | --- | --- | --- | --- | --- | --- |
| SWMM | 3.9 | 7.3 | 6.2 | 4.0 | 5.8 | 10.1 | 4.9 |
| GAST | 2.2 | 2.8 | 5.5 | 2.8 | 3.5 | 7.8 | 0.74 |

**The data in Figure 10 (a)**

| Time (minutes) | 200mm (mm/h) | 500a(mm/h) | 200a(mm/h) | 100a(mm/h) | 50a(mm/h) |
| --- | --- | --- | --- | --- | --- |
| 0 | 291.6 | 154.0 | 165.6 | 130.8 | 72.7 |
| 20 | 477.4 | 268.4 | 222.0 | 154.0 | 154.0 |
| 40 | 1102.6 | 628.3 | 523.8 | 477.4 | 396.1 |
| 60 | 3822.5 | 2502.3 | 2202.2 | 2016.4 | 1843.9 |
| 80 | 1611.8 | 905.3 | 686.3 | 651.5 | 581.9 |
| 100 | 800.8 | 465.8 | 372.9 | 338.1 | 268.4 |
| 120 | 419.3 | 268.4 | 256.8 | 210.4 | 165.6 |
| 140 | 407.7 | 222.0 | 142.4 | 130.8 | 107.5 |

**The data in Figure 10 (b)**

| Scenario Type | Proportion (%) |
| --- | --- |
| Scenario 1 | 4.5 |
| Scenario 2 | 5.7 |
| Scenario 3 | 7.1 |
| Scenario 4 | 8.8 |
| Scenario 5 | 11.3 |

**The data in Figure 11 (a)**

| Node Degree | Number of Nodes |
| --- | --- |
| 1 | 14.44 |
| 2 | 10.01 |
| 3 | 51.94 |
| 4 | 43.75 |
| 5 | 17.78 |

**The data in Figure 11 (b)**

| Network Diameter | Probability Distribution | Gaussian Fit |
| --- | --- | --- |
| 0 | 0.021 | 0.023 |
| 4 | 0.078 | 0.075 |
| 8 | 0.091 | 0.101 |
| 12 | 0.075 | 0.078 |
| 16 | 0.035 | 0.036 |
| 20 | 0 | 0 |

**The data in Figure 12 (a)**

| Node Degree | Scenario 2 | Scenario 5 |
| --- | --- | --- |
| 1 | 15.7 | 25.1 |
| 2 | 30.1 | 30.4 |
| 3 | 45.5 | 35.1 |
| 4 | 15.3 | 10.4 |

**The data in Figure 12 (b)**

| Network Diameter | Gaussian Fit | Probability Distribution |
| --- | --- | --- |
| 0 | 0 | 0 |
| 4 | 0.031 | 0.031 |
| 8 | 0.099 | 0.101 |
| 12 | 0.120 | 0.121 |
| 16 | 0.081 | 0.082 |
| 20 | 0.028 | 0.029 |

**The data in Figure 12 (c)**

| Network Diameter | Gaussian Fit | Probability Distribution |
| --- | --- | --- |
| 0 | 0.030 | 0.021 |
| 6 | 0.049 | 0.052 |
| 12 | 0.055 | 0.051 |
| 18 | 0.031 | 0.035 |
| 24 | 0.014 | 0.017 |
| 30 | 0.004 | 0.001 |

**The data in Figure 14(a)**

| Scenario | Dikstra (s) | SPFA (s) | Floyd-Warshall (s) |
| --- | --- | --- | --- |
| 1 | 421 | 338 | 415 |
| 2 | 521 | 443 | 478 |
| 3 | 715 | 684 | 861 |
| 4 | 872 | 665 | 891 |
| 5 | 915 | 875 | 1000 |

**The data in Figure 14 (b)**

| Scenario | SPFA (km) | Floyd-Warshall (km) | Dijkstra (km) |
| --- | --- | --- | --- |
| 1 | 6021.0 | 6040.4 | 6195.5 |
| 2 | 6311.2 | 6510.0 | 6829.3 |
| 3 | 6790.5 | 7032.2 | 7129.2 |
| 4 | 6969.9 | 7177.7 | 7506.7 |
| 5 | 7618.2 | 7748.4 | 8087.8 |

**The data in Figure 16 (a)**

| Scenario Type | LQR-RRT(m) | A*(m) | DP(m) |
| --- | --- | --- | --- |
| Scenario 1 | 7255.8 | 7324.5 | 7255.8 |
| Scenario 2 | 7680.9 | 7935.2 | 7859.6 |
| Scenario 3 | 7694.7 | 8154.2 | 7996.1 |
| Scenario 4 | 7763.4 | 8264.1 | 8167.9 |
| Scenario 5 | 8037.3 | 8504.7 | 8792.3 |

**The data in Figure 16 (b)**

| Scenario Type | LQR-RRT(s) | A*(s) | DP(s) |
| --- | --- | --- | --- |
| Scenario 1 | 724.7 | 765.9 | 753.3 |
| Scenario 2 | 777.4 | 849.3 | 827.7 |
| Scenario 3 | 821.9 | 896.3 | 855.0 |
| Scenario 4 | 912.1 | 991.1 | 969.3 |
| Scenario 5 | 1049.4 | 1100.8 | 1183.2 |
